# Evaluating time-reversed speech and signal-correlated noise as auditory baselines for isolating speech-specific processing using fNIRS

**DOI:** 10.1371/journal.pone.0219927

**Published:** 2019-07-17

**Authors:** Faizah Mushtaq, Ian M. Wiggins, Pádraig T. Kitterick, Carly A. Anderson, Douglas E. H. Hartley

**Affiliations:** 1 National Institute for Health Research Nottingham Biomedical Research Centre, Nottingham, United Kingdom; 2 Hearing Sciences, Division of Clinical Neuroscience, School of Medicine, University of Nottingham, Nottingham, United Kingdom; 3 Nottingham University Hospitals NHS Trust, Queens Medical Centre, Nottingham, United Kingdom; Preeminent Medical Phonics Education & Research Center, Hamamatsu University School of Medicine, JAPAN

## Abstract

Evidence using well-established imaging techniques, such as functional magnetic resonance imaging and electrocorticography, suggest that speech-specific cortical responses can be functionally localised by contrasting speech responses with an auditory baseline stimulus, such as time-reversed (TR) speech or signal-correlated noise (SCN). Furthermore, these studies suggest that SCN is a more effective baseline than TR speech. Functional near-infrared spectroscopy (fNIRS) is a relatively novel, optically-based imaging technique with features that make it ideal for investigating speech and language function in paediatric populations. However, it is not known which baseline is best at isolating speech activation when imaging using fNIRS. We presented normal speech, TR speech and SCN in an event-related format to 25 normally-hearing children aged 6–12 years. Brain activity was measured across frontal and temporal brain areas in both cerebral hemispheres whilst children passively listened to the auditory stimuli. In all three conditions, significant activation was observed bilaterally in channels targeting superior temporal regions when stimuli were contrasted against silence. Unlike previous findings in infants, we found no significant activation in the region of interest over superior temporal cortex in school-age children when normal speech was contrasted against either TR speech or SCN. Although no statistically significant lateralisation effects were observed in the region of interest, a left-sided channel targeting posterior temporal regions showed significant activity in response to normal speech only, and was investigated further. Significantly greater activation was observed in this left posterior channel compared to the corresponding channel on the right side under the normal speech vs SCN contrast only. Our findings suggest that neither TR speech nor SCN are suitable auditory baselines for functionally isolating speech-specific processing in an experimental set up involving fNIRS with 6–12 year old children.

## Introduction

Speech processing in the brain is complex, comprised of multiple parallel and hierarchical processing streams, occurring in several phases across different areas within the brain [1]. Undoubtedly, good speech and language skills are important for effective social functioning, proficient literacy abilities, a successful education and even maintaining a job [2]. Therefore, it is clinically useful to isolate speech-specific activity within the auditory cortex that is responsible for processing higher-level linguistic aspects of speech, rather than more general lower-level acoustic information, particularly at an individual level. This is important, for example, when investigating atypical language profiles [3, 4] when examining the neural substrates of different phonological units of speech [5], for mapping of cortical functions prior to surgery [6–8] and to identify successful speech recognition in hearing impaired populations [9, 10].

Since the emergence of functional neuroimaging techniques, it has become easier to localise cortical areas responsible for speech-specific processing. It is known, for example, that the acoustic features of speech are largely processed in the primary auditory cortex [1] and there is substantial evidence to suggest that the left hemisphere (LH) plays the principal role in speech and language processing in approximately 90% of the population [11–13], with this specialisation present even at birth [14, 15]. Therefore, it is possible that left-lateralised responses to speech could be taken as a proxy for normal speech-related brain organisation and function. The extent of left-sided lateralisation of brain activity in response to different auditory inputs could be used to determine whether a child is not only receiving speech signals but that their brain is registering these signals as speech. However, although the auditory system is well tuned towards the acoustic characteristics of speech [1], attempting to isolate parts of the cortex that respond specifically to speech in order to explore language processing in the brain is challenging [16]. Indeed, cortical activations to linguistic elements of speech are tightly packed together with primary auditory responses within superior temporal regions that compounds this issue of isolating speech-specific processing [1].

As suggested by Stoppelman et al. [16], one possible solution is to contrast brain responses to speech against activity elicited by an auditory baseline so that speech-specific activity can be isolated. The best auditory baseline is one that can isolate responses to speech from other cognitive or auditory processes [16]. In order to achieve this, the baseline must have identical acoustic, but not linguistic, properties to speech, which is problematic since prosody and phonology are acoustically defined linguistic characteristics of speech [16]. Essentially, a good speech baseline is one which is as similar to normal speech as possible without being normal speech. Two commonly used baselines are time-reversed (TR) speech and signal-correlated noise (SCN) [14–21].

TR speech is an unintelligible speech stimulus in which the universal features of normal speech such as voicing, segmentation of words and articulatory characteristics are preserved [16] whilst onsets are slower and decays are more rapid than normal speech [22]. The reversal of the speech breaks down phrase and sentence prosody along with the phonotactic composition of the speech, generating utterances that cannot be vocalised [22]. SCN, on the other hand, is a non-speech noise signal comprised of speech-shaped noise that has been modulated by the amplitude envelope extracted from the original speech signal. Speech-like rhythmic onsets are preserved in SCN but other characteristics of speech such as phonemic structure and pitch are lost, making SCN completely unintelligible [16, 20, 23, 24].

When brain responses to normal speech are contrasted against responses to TR speech and SCN, using functional magnetic resonance imaging (fMRI) for example, activation in the primary auditory cortex is removed in both instances [16]. However, a large proportion of the activity in language areas is also removed in the normal speech vs TR speech contrast as responses to both these stimuli appear to overlap significantly [16, 20]. Nonetheless, TR speech remains a popular control for speech processing, particularly with younger populations, and has shown to elicit weaker responses than normal speech in fMRI studies in the temporal cortex as well as in parietal regions [25].

Unfortunately, many traditional neuroimaging techniques used to investigate speech and language processing are not always suitable for testing certain study populations. For example, subjects are required to remain still within MRI scanners in order to minimise movement noise in the data. With young children and babies, this may require sedation or anaesthesia, which, as well as being more risky, may also influence functional brain responses, particularly to speech. In addition, MRI scanners are often noisy, which makes measuring responses to auditory stimuli challenging. Measuring brain activity in individuals who have certain implanted devices which cannot be easily removed and contain magnetic and/or electronic components, such as cochlear implants, can interfere with the recordings and corrupt the data using a number of imaging modalities including fMRI, electroencephalography and magnetoencephalography [26]. Other techniques, such as positron emission tomography, risk exposing a subject to radiation which limits the number of times an individual can be scanned [26]. Arguably these issues are more of a concern when the study involves irradiation exposure of babies or children.

Similar to fMRI, fNIRS is also based on neurovascular coupling principles [27]. Cerebral functionality is investigated by measuring changes in concentrations of oxygenated haemoglobin (HbO), deoxygenated haemoglobin (HbR) and total haemoglobin and their timing with stimuli (e.g., auditory input) [27], enabling an indirect measure of neuronal activity. Unlike other imaging techniques, fNIRS is an optically-based brain imaging technique that is non-invasive, safe, portable and relatively inexpensive [28]. These factors make fNIRS ideal for use not only in research settings, but in clinical settings as well since techniques with low running costs, no disposables and short imaging times can be more readily integrated into clinical pathways. Furthermore, portability allows for patients, especially younger individuals, to be scanned in more comfortable environments, with the option of changing locations with ease if necessary. fNIRS is relatively insensitive to head movements and allows for various head positions and postures as the optical fibres are flexible [29]. This is makes it particularly useful for imaging babies and young children as they are not required to keep very still, be placed within a scanner or confined space, or undergo restraint or sedation [30]. Since fNIRS does not involve exposing subjects to radiation, it is safe for repeated use [26] and scanning is silent, which is particularly important when presenting auditory stimuli and investigating auditory brain responses [31]. Furthermore, fNIRS is fully compatible for use with individuals who have been fitted with implantable prostheses, such as a cochlear implant, and data collected using fNIRS is not affected by any magnetic and electronic parts in these devices [26].

The primary aim of this study was to identify an appropriate functional baseline for speech-specific processing using fNIRS in normal hearing (NH) school-age children. We compared two commonly used auditory baselines for speech processing in functional neuroimaging studies: TR speech and SCN [14–21]. Previous work has been conducted to help clarify which of these two stimuli offer the better contrast against normal speech when attempting to functionally isolate speech-specific cortical activity in temporal and frontal regions. Often, SCN is favoured over TR speech, but this work has only involved fMRI and electrocorticography [16, 20, 21] and has not yet been explored with fNIRS.

## Methods

### Participants

Twenty-five children (mean age 8.8 years; age range 6–12 years; 10 males) participated in the study. Participants were primarily recruited via posters and online adverts. All children were native English speakers with normal or corrected-to-normal vision, no known hearing problems, and no history of cognitive or motor impairment. All participants passed a pure tone audiometry air-conduction hearing screen performed at 20 dB HL at 1, 2, 4 and 0.5 kHz respectively in both ears (procedure adapted from the British Society of Audiology [32]). All participants also scored 100% on a speech perception assessment during which they were asked to listen to and repeat a set of sixteen sentences and were scored against fifty pre-determined keywords [33]. An example sentence with the keywords underlined is: He played with his train. Nineteen children were right handed as assessed using a motor-speech laterality questionnaire by Flowers and Hudson [34]. Intelligence was assessed using the Wechsler Abbreviated Scale of Intelligence–Second Edition (WASI-II) [35, 36] with the group average age-corrected intelligence quotient (IQ) ranked at the 58^th^ percentile (range 14^th^ to 95^th^ percentile). Written informed consent was obtained from the accompanying parents or guardians of all participants. Participants were also required to give verbal assent. The study was approved by the University of Nottingham Faculty of Medicine and Health Sciences Research Ethics Committee.

### Test procedure

In an event-related design, participants were presented with three auditory stimulus conditions: normal speech, TR speech and SCN. A total of 25 different sentences were played at random per condition, with an additional 25 sentences muted for a silent condition. The presentation of each sentence lasted 1.64 s on average (range 0.86 s to 2.30 s). The stimulus onset asynchrony (the time between the onset of one sentence and the next) was varied randomly in the range 2.5 s to 5.0 s. Jittering the stimulus onset asynchrony across trials has been shown to improve the efficiency of event-related experiments [37] and has been used in our previous work [10, 38]. It helps to reduce the influence of preparatory and anticipatory factors and enables responses to different conditions to be deconvolved despite the temporal overlap in the haemodynamic activity elicited by successive trials [37]. In order to encourage the young participants to attend to the auditory stimuli a warble tone was presented at random 12 times throughout the test. Subjects were instructed to listen carefully to the auditory stimuli and press a button on a response box (‘RTbox’) [39] as quickly as possible whenever this tone was heard. Reward stars provided additional encouragement. Specifically, at five evenly spaced intervals throughout the experiment, participants could track their progress through the experiment by counting stars that were displayed on a visual display unit for 4 s: one star representing each fifth of the experiment that participants had completed. When the reward stars were not displayed on the screen a plain grey background was shown with a small fixation cross in the centre which participants were instructed to look at. Note that responses to the attention trials and reward stars were only included as regressors of no interest in the analysis. The fNIRS imaging lasted approximately 8 minutes in total. Prior to the placement of the optode array on the subject’s head and the start of the fNIRS imaging measurements, participants completed a short practice session in order to become familiar with the task and stimuli.

### Equipment

Testing was conducted within a sound-treated room with dimmed lighting. Participants were seated comfortably at a distance of approximately 75 cm from a visual display unit above which a loudspeaker was positioned (Model 8030A, Genelec, Iisalmi, Finland). Auditory stimuli were presented from the speaker in the free-field. Although fNIRS recordings are relatively silent, a sound absorbing screen was positioned between the fNIRS equipment and the participant to render noise from the equipment inaudible.

Brain activity was measured non-invasively using a continuous wave fNIRS system (ETG-4000, Hitachi Medical Co., Japan). This system minimizes crosstalk between wavelengths and channels using frequency modulation [40]. Thirty optodes were arranged in two 3 x 5 arrays with a fixed source-detector gap of 3 cm. Responses were measured concurrently from both cerebral hemispheres from a total of 44 measurement channels at wavelengths of 695 nm and 830 nm (sampling rate 10 Hz). The experiment was programmed in MATLAB (Mathworks, Natick, MA) using the Psychtoolbox-3 extensions [41–43].

**Fig 1:**
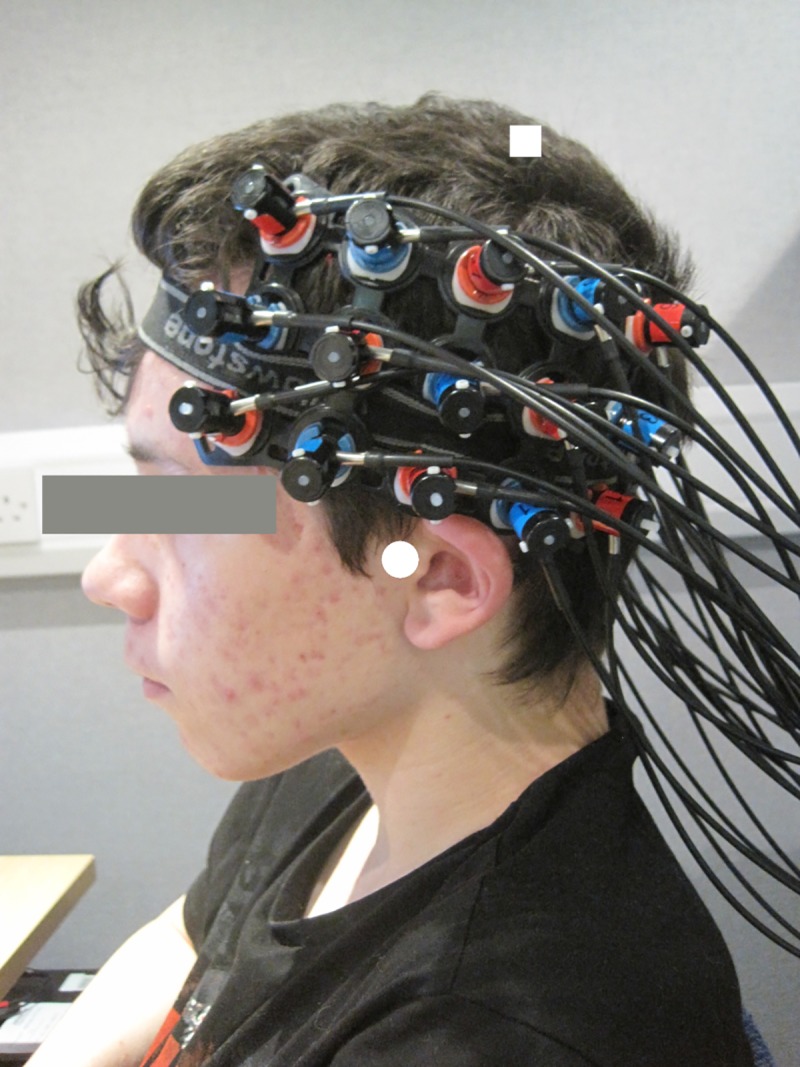
Typical optode array placement. Photograph of typical optode array placement on a volunteer’s head (consent obtained for use of photograph). The white square indicates point Cz and the white circle indicates the preauricular point, as taken from the International 10–20 system to guide array placement.

The optode array was placed on the participant’s head over bilateral temporal and frontal brain regions in order to provide sufficient coverage for patterns of hemispheric laterality to be investigated. The International 10–20 positioning system was used as a guideline [44] so as to ensure consistent array placement across participants. The middle optode on the bottom row was positioned as close to the preauricular point as possible and the middle optode on the top row was directed towards point Cz, as shown in Fig 1. (The parent of the participant in Fig 1 has given written informed consent (as outlined in PLOS consent form) to publish their photograph.) If necessary, hair was moved out of the way from underneath optodes to maximise contact with the scalp using a small illuminated tool. Once the position of the optode array was completed, a photograph was taken of the final placement for reference purposes. During testing, participants were instructed to remain still and keep head movements to a minimum to reduce motion artefacts in the recorded data.

### Speech stimuli

Recordings of a male speaker reciting Bamford-Kowal-Bench sentences (BKB) [33] were used as auditory stimuli during the fNIRS measurements and for the speech perception assessment. A total of twenty lists were available, each containing sixteen sentences. For the fNIRS task, one hundred sentences were chosen at random to form the three speech conditions and the silent condition. For the speech perception assessment, one list was selected at random from lists that had not already been selected for use in the fNIRS task. Speech stimuli were presented at a level of 65 dB SPL (A-weighted root-mean-square level averaged over the duration of each sentence) measured at the participant’s listening position with the participant absent using a sound level meter (Type 2250, Brüel & Kjær, Nærum, Denmark).

For the TR speech condition, the audio signal was reversed so that the sentence was played backwards. For the SCN condition, a fast Fourier transform of the original speech signal was performed. Following this, the phase information was randomised while retaining the magnitude spectrum. This resulted in the removal of all of the temporal information in the original speech whilst preserving the distribution of energy across frequencies. After conversion back to the time domain, the signal was then modulated by a low-pass (50 Hz) filtered envelope extracted from the original sentence using the Hilbert transform. All speech stimuli were processed using MATLAB.

### fNIRS data analysis

The fNIRS measurements were analysed in MATLAB using functions from the HOMER2 package [45] alongside custom scripts developed in our lab and used in our previous work [10, 38, 46–48]. After the raw fNIRS intensity signals had been converted into changes in optical density, motion artefact correction was conducted with a wavelet filtering technique applied using the HOMER2 *hmrMotionCorrectionWavelet* function [49]. This function eliminates outlying wavelet coefficients which are assumed to be motion artefacts by implementing a probability threshold. We chose to omit the coefficients which lay more than 0.719 times the interquartile range below the first or above the third quartiles. If the wavelet coefficients are assumed to be normally distributed, this equates to the α = 0.1 threshold used in fNIRS motion artefact correction method evaluations [50, 51].

Next, the data were bandpass filtered between 0.02 and 0.5 Hz in order to attenuate cardiac oscillations and low frequency drift. The optical density signals were then converted into estimates of HbO and HbR using the modified Beer-Lambert law [45]. At both wavelengths a default value of 6 was used for the differential path-length factor. Since we were interested in contrasting relative responses across conditions rather than estimating absolute changes in haemoglobin concentrations, we did not account for the partial volume effect linked to focal haemodynamic changes [52].

The signal separation algorithm described by Yamada et al. [53] was used to isolate the functional component of the haemodynamic signal. In this algorithm, the impact of systemic physiological signals is decreased by making use of the negative correlation between changes in HbO and HbR concentrations in functional cerebral responses and, conversely, the positive correlation between HbO and HbR concentration changes elicited by systemic physiological oscillations and head movements [53]. As demonstrated in our previous work, at a group level, the use of this algorithm results in an improvement in the reliability of fNIRS responses [47].

Finally, although every effort was made to obtain good contact between the scalp and the optodes, the fNIRS data were reviewed before any statistical analyses were performed. In order to remove any channels with poor signal quality, the scalp coupling index (SCI) method by Pollonini et al. [54] was administered. The fNIRS data at the two wavelengths were bandpass filtered between 0.5 and 2.5 Hz in order to separate the cardiac element with the degree of correlation between the two wavelengths taken as an indicator of how well the optodes had contacted with the scalp. We chose to exclude the worst 5% of channels from the data (SCI threshold of ≥ 0.13). This was deemed appropriate so that as many channels as possible could be preserved and used for statistical analyses, especially since the optode array did not allow for spatially overlapping channels.

In order to conduct statistical analyses, the general linear model approach was adopted to calculate the haemodynamic response amplitude on a channel-wise basis [55]. A set of 3 regressors for each of the speech conditions as well as an extra set for the silent condition were included in the design matrix. Each individual trial was modelled as an epoch corresponding to the stimulation duration. The time courses were then convolved with the canonical haemodynamic response function (HRF) provided in SPM8 [http://www.fil.ion.ucl.ac.uk/spm]. The first temporal derivative and the second temporal derivative (the dispersion derivative) of the canonical were also included to enable the model to recognise responses with longer activation durations than that of the canonical HRF or those which had shifted in time [38, 56–58]. For each condition, the regressor relating to the temporal derivative was orthogonalized with respect to the canonical HRF regressor, and the regressor relating to the dispersion derivative was orthogonalized with respect to both the canonical HRF regressor and its temporal derivative regressor [38]. Two additional sets of three regressors-of-no-interest corresponding to the attentional warble tone trials and the progress stars were also included in the analysis. This was done to ensure that brain activity relating to these was appropriately captured by the model even though the resulting estimates were not of interest. Model estimation was conducted using a dual-stage ordinary least squares procedure [59] with serial correlation accounted for using the Cochrane-Orcutt technique [60].

To quantify the strength of the haemodynamic response in a way that would be minimally affected by any differences in response latency or dispersion between conditions, we used the ‘derivative-boost’ technique [61] to calculate the ‘estimated response amplitude’ (ERA). The derivative-boost technique combines the beta weights corresponding to the three regressors for each condition (the canonical HRF and its temporal and dispersion derivatives) as follows:

$$ERA=sign({\hat{\beta }}_{1})\times \sqrt{{{\hat{\beta }}_{1}}^{2}+{{\hat{\beta }}_{2}}^{2}+{{\hat{\beta }}_{3}}^{2}}$$

where ${\hat{\beta }}_{1},\;{\hat{\beta }}_{2}$ and ${\hat{\beta }}_{3}$ are the estimated beta weights for the canonical, temporal derivative and dispersion derivative terms, respectively. To ensure correct scaling of the regressors when computing the ERA, we post-normalised the relevant columns of the design matrix as described by Steffener et al. [62]. Significant cortical activation was tested for using one-sided *t*-tests (α level 0.05) at a group level (random-effects analysis). The contrasts investigated were: (i) each auditory condition vs silence and (ii) normal speech vs the two unintelligible speech conditions (TR speech and SCN). We used one-sided tests since we had clear directional hypotheses that: i) all acoustic stimuli would elicit positive activation compared to silence and ii) activation would be greater in response to normal speech than to both unintelligible baseline stimuli. To evaluate these contrasts, the ERAs for the relevant conditions were subtracted one from the other. In order to take into account the matter of multiple comparisons due to testing for significant activation at each channel separately, the false discovery rate (FDR) technique described by Benjamini and Yekutieli [63] was adopted.

In addition to performing map-wise analyses across the full optode array, we extracted ERAs, and evaluated the associated contrasts, from our specific region of interest (ROI) which was defined based on our previous work with adults [10]. Channels covering auditory regions within the superior temporal cortex in both cerebral hemispheres were selected. These were channels 29 and 33 in the LH and channels 7 and 12 in the right hemisphere (RH). Single-subject level responses for each channel were used in repeated measures analyses of variance (RM- ANOVAs), performed using IBM SPSS Statistics for Windows Version 24.0 software (IBM Corp., Armonk, New York). The first within-subject factor was “contrast” which had two levels (normal speech vs TR speech and normal speech vs SCN) or three levels (normal speech vs silence, TR speech vs silence, SCN vs silence) and the second was “brain hemisphere” which had two levels (left-sided or right-sided channels).

### Laterality assessment

Hemispheric dominance is often indicated by a laterality index, calculated using the following formula: (Q_LH_—Q_RH_) / (Q_LH_ + Q_RH_) where Q_LH_ and Q_RH_ are representative quantities measured in some way (e.g., fMRI) for the contributions from the LH and RH, respectively [64]. The resultant value usually ranges between -1 (pure RH dominance) and +1 (for pure LH dominance). However, this formula only applies if all measures are a positive value, which was not the case in our data. Therefore, we calculated activity lateralised to the LH by subtracting right-sided ERAs from left-sided values for each (i) auditory stimulus vs silence contrast and each (ii) normal speech vs baseline contrast. Although we anticipated left-hemispheric dominance for speech, in map-wise analyses we compared LH and RH responses using two-sided statistical tests to allow for the possibility of right-lateralised activation.

## Results

### Data pre-processing

Usable data were obtained from twenty-three out of the twenty-five participants tested. Data from the remaining two participants were rendered unusable by pronounced movement artefacts or measurement artefacts, attributed to problems with poor optode-scalp contact.

### Experimental condition contrasts

Initially, the most pronounced contrasts, between the auditory stimuli and silence, were investigated to confirm that the expected effects were present in the most rudimentary contrast to form a justified basis for further analysis of the data containing subtler contrasts. Furthermore, significant differences between activity elicited by auditory stimuli and silence would confirm that successful fNIRS measurements had been taken. Following this, analyses were conducted to examine differences in activation elicited between normal speech vs TR speech and SCN.

**Fig 2:**
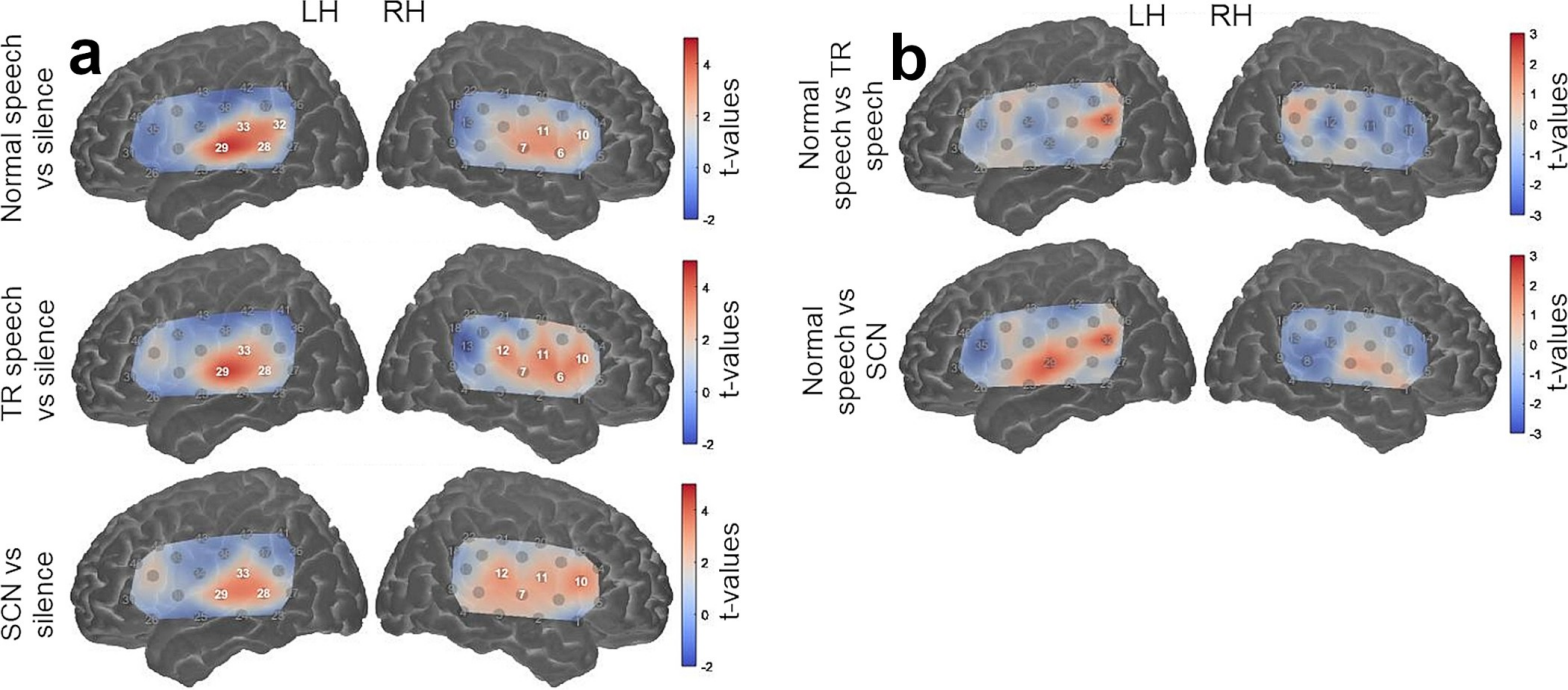
Group level cortical activation maps for each experimental contrast in the LH and the RH. Highlighted channels show significant activation (q < .05, FDR corrected). (a) Shows responses to the three auditory conditions (normal speech, TR speech and SCN) contrasted against silence. (b) Shows responses to normal speech contrasted against the two auditory baselines (TR speech and SCN). Note that the maps are interpolated from single-channel results and the overlay on the cortical surface is for illustrative purposes only.

**Fig 3:**
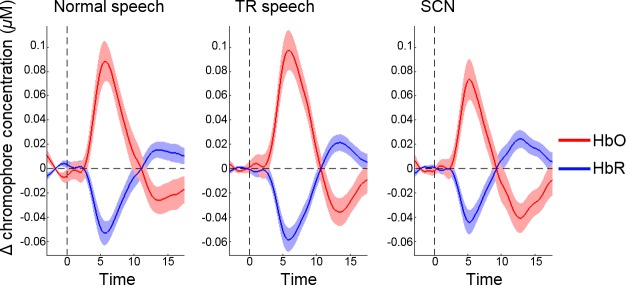
Block-averaged haemodynamic time courses. These are displayed for each type of auditory stimulus (response to silent trials subtracted out). Responses were averaged across channels 28, 29 and 33 (left hemisphere) and channels 7, 10 and 11 (right hemisphere) targeting the superior temporal cortex.

Activation maps for each auditory stimulus condition contrasted against the silent baseline at a group level are shown in Fig 2A. In all three conditions, statistically significant activation (*q* < 0.05, FDR corrected) was observed in both hemispheres in channels targeting the auditory cortices, with a visual inspection indicating a greater spread of activation within the RH compared to the LH. The significantly activated channels common in all three auditory stimulus conditions (when contrasted against silence) were channels 28, 29 and 33 in the LH and channels 7, 10 and 11 in the RH. Block-averaged haemodynamic time courses derived from these six channels are shown in Fig 3. Activation maps for normal speech against TR speech and SCN are shown in Fig 2B. At a group level, no channels showed significant activation (*q* < 0.05, FDR corrected) for either contrast.

### Laterality assessment

**Fig 4:**
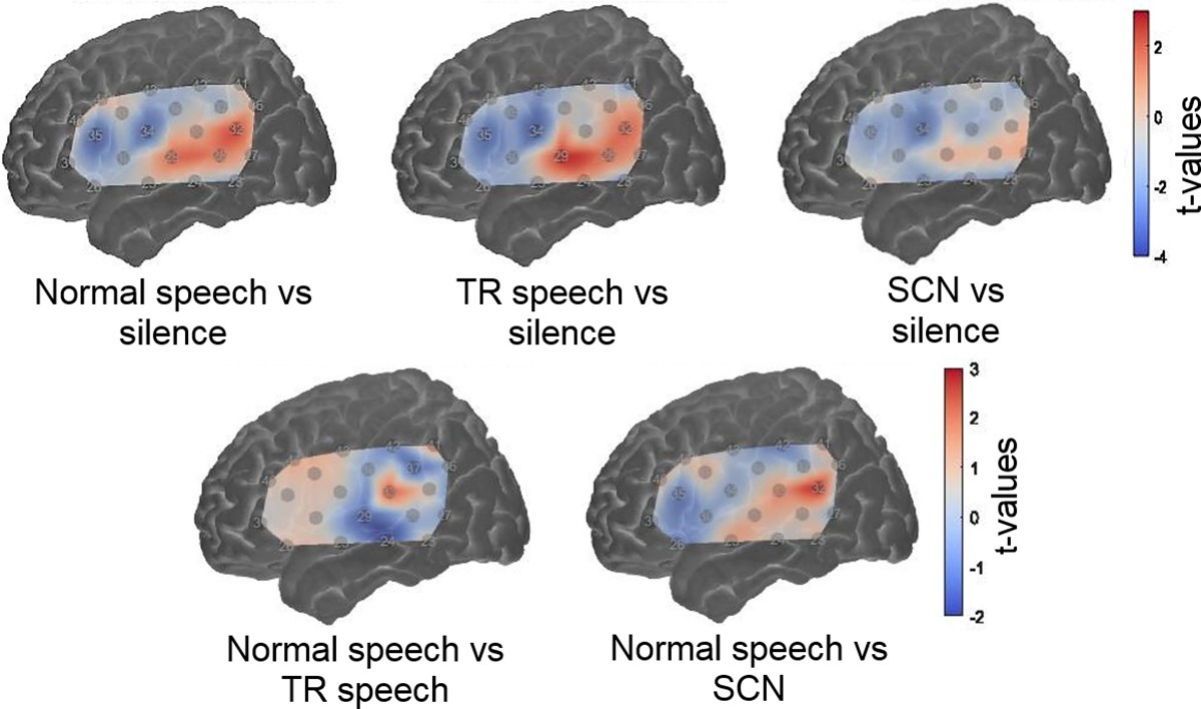
Group level cortical activation maps for each experimental contrast showing LH—RH activity. Results are shown projected on to the left hemisphere. Positive *t*-values indicate greater activity in LH channels compared to the corresponding channels in the RH. Negative *t*-values indicate that RH activity was greater. No channels showed a significant hemispheric difference (*q* < .05, FDR corrected).

Group level activation maps displaying activation lateralised to the LH are shown in Fig 4. No statistically significant difference in activation between the two hemispheres was observed in any channel under any condition contrast (*q* < 0.05, FDR corrected).

### ROI statistical analyses

ROI statistical analyses were conducted to examine differences in activity specific to auditory regions within the superior temporal cortex. This a priori ROI was comprised of channels 29 and 33, targeting the LH, and channels 7 and 12, targeting the RH. An RM-ANOVA was conducted using the ERAs for the three auditory condition contrasts against silence (normal speech vs silence, TR speech vs silence and SCN vs silence) from the channels in the ROI. There was no main effect of brain hemisphere (F(1, 22) = 1.533, *p* > .05) or contrast (F(2, 44) = 1.591, *p* > .05). There was also no significant interaction between the two (F(1.328, 29.210) = 1.871, *p* > .05).

**Fig 5:**
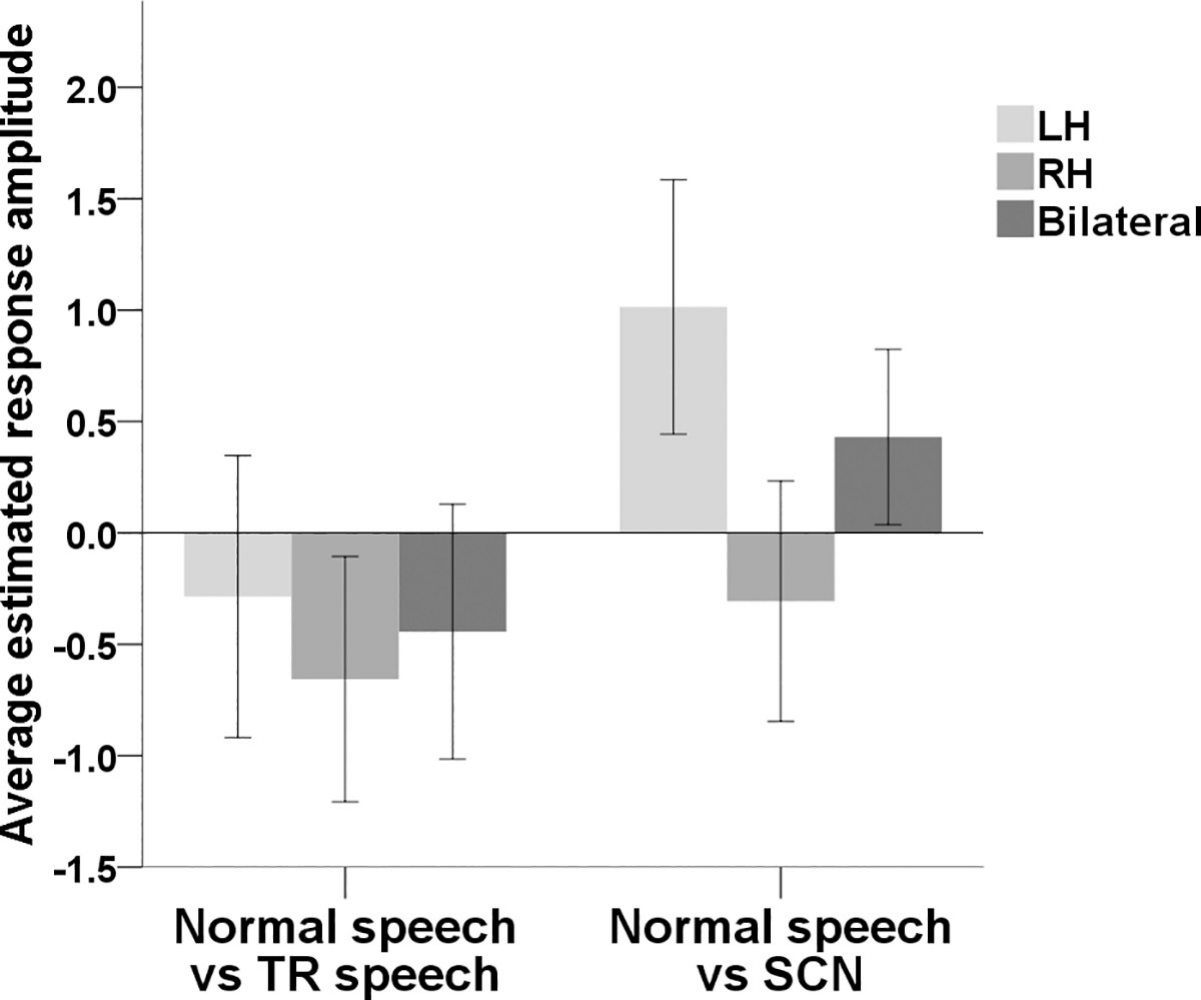
Mean ERAs (N = 23) for each auditory condition contrast derived from the ROI. Mean ERAs are shown for auditory regions in the LH (channels 29 and 33) and the RH (channels 7 and 12). Bilateral ERAs (average across all four of these channels) are also shown. Error bars show ±1 standard error of the mean.

A second RM-ANOVA was conducted using the group average ERAs for the auditory condition contrasts (normal speech vs TR speech and normal speech vs SCN) for the ROI. Fig 5 shows average ERAs for each contrast for the pre-selected channels in the LH, RH and bilaterally in the ROI. Once again, there was no statistically significant main effect of brain hemisphere (F(1, 22) = 3.228, *p* > .05) or contrast (F(1, 22) = 3.731, *p* > .05). Again, there was no significant interaction between the two (F(1, 22) = 1.295, *p* > .05).

In order to confirm the null results were not due to the specific channels included in our a priori ROI, a secondary post hoc analysis using another ROI was performed using the group average ERAs for the two auditory condition contrasts. This secondary ROI was derived from the common activation pattern elicited by TR speech and SCN contrasted against silence. Although this data-driven ROI overlapped considerably with our a priori ROI, a number of additional channels were included in the analysis (channels 10, 11 and 28). A null result was similarly obtained with no main effect of brain hemisphere (F(1, 22) = 2.833, p > .05), contrast (F(1, 22) = 3.919, p < .05) or interaction between the two observed (F(1, 22) = .350, p > .05).

### Additional analyses in posterior auditory regions

**Fig 6:**
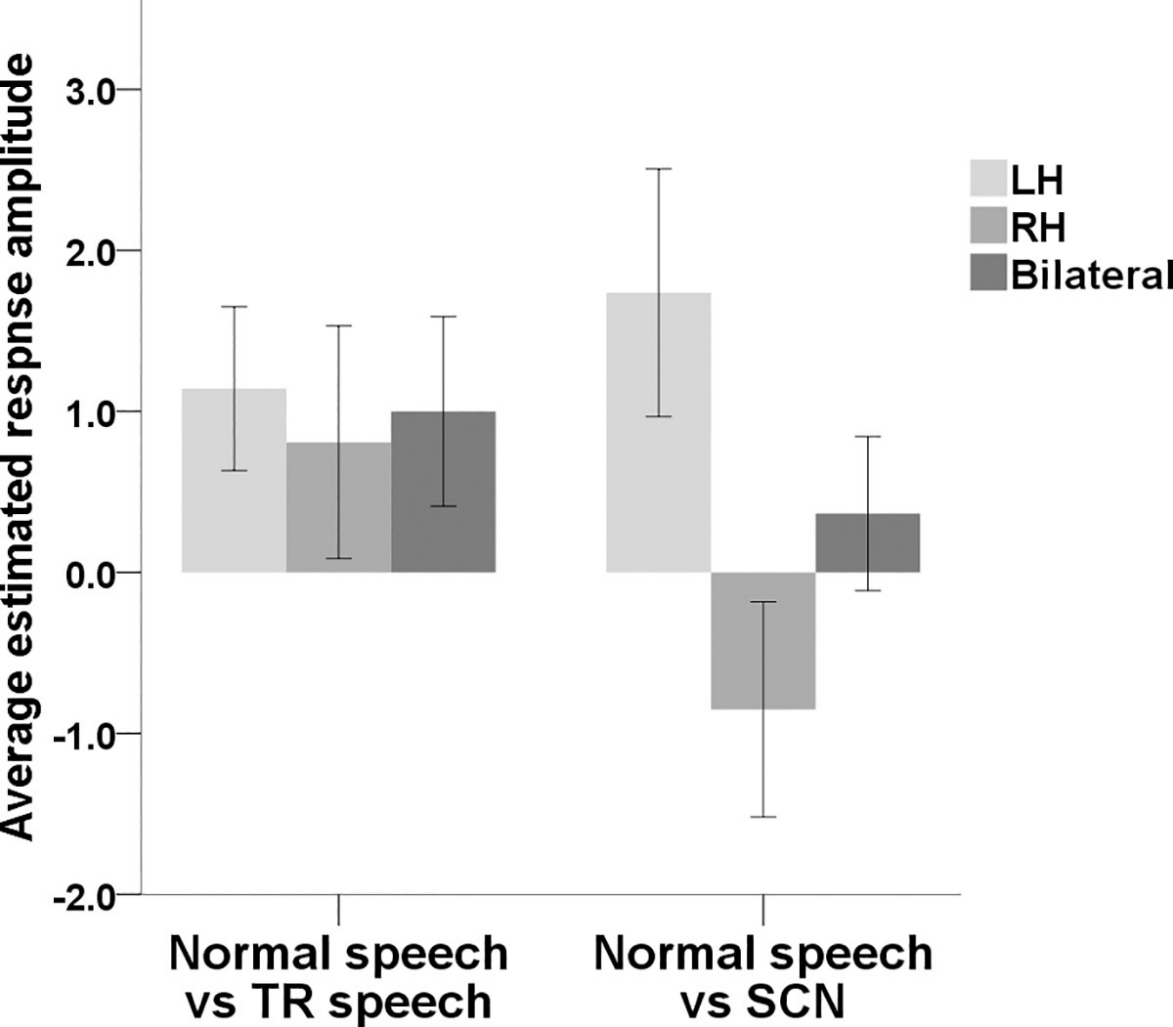
Mean ERAs (N = 20) for each auditory condition contrast derived from posterior temporal regions. Mean ERAs are shown for channels 32 and 13, targeting posterior temporal regions in the LH and RH respectively. Bilateral ERAs (average across these two channels) are also shown. Error bars show ±1 standard error of the mean.

Interestingly, channel 32, which targeted posterior superior temporal regions in the LH, was the only channel, as displayed in Fig 2A, to show significant activation in response to intelligible speech but not to either of the unintelligible controls (when contrasted against silence). Although this was not an area we had a priori predictions regarding, it was deemed beneficial to investigate it further considering the exploratory nature of the study. Therefore, ERAs for the auditory condition contrasts (normal speech vs TR speech and normal speech vs SCN) from channel 32 and the corresponding channel on the right side (channel 13) were used to run an RM-ANOVA analysis. The data pre-processing procedure resulted in the exclusion of 3 participants from this analysis (N = 20). Two participants were excluded as their data from channel 13 was of poor quality and the third participant was excluded as their data from channel 32 was of poor quality. Average ERAs for each contrast in the LH, RH and bilaterally are displayed in Fig 6. There was a statistically significant main effect of brain hemisphere (F(1, 19) = 5.657, *p* < .05) but no significant main effect of contrast (F(1, 19) = .799, *p* > .05). There was also a significant interaction between the two (F(1, 19) = 5.248, *p* < .05). In order to investigate the interaction further, a paired samples *t*-test was carried out. Whilst there was no significant difference between the normal vs TR speech contrast average ERAs in the left vs the right hemispheres (*t*_19_ = -.724, *p* > 0.05), there was a statistically significant difference between the two hemispheres when brain activation to normal speech was contrasted against SCN (*t*_19_ = 2.635, *p* < .05).

## Discussion

It is important to study potential limitations of novel neuroimaging tools, such as fNIRS, to measure speech activation in typically developing children. We contrasted normal speech against TR speech and SCN to determine which auditory baseline is more suitable for functionally isolating responses to intelligible speech in a paediatric population when measuring cortical activation using fNIRS. Although we successfully measured brain activation in response to auditory stimuli within the auditory cortices of 23 NH children, there was no statistically significant difference between the brain activity elicited by normal speech contrasted against the unintelligible speech stimuli we used or between responses in the LH and RH. This suggests that neither TR speech nor SCN are effective baselines for isolating speech-specific activity or measuring lateralised responses using fNIRS in NH children aged 6–12 years.

Although a number of studies have identified differences in cortical responses to TR speech and SCN in infants [14, 15, 65, 66] and adults [16, 67, 68], our population consisted of healthy children aged 6–12 years, a period of rapid growth of the skull and the brain [69, 70]. These individual variations in head growth could have considerable impact on data collected from neuroimaging techniques [71]. For example, there is a large degree of individual variability in the total surface area of a flattened cerebral cortex which increases non-linearly during the first decade of life before going on to decrease until approximately 20 years of age [72]. Average cortical thickness, on the other hand, decreases from age 3 to 20 years in a much more linear fashion [72].

These complex differences between children of the same age and across developmental stages can interfere with interpretations of observed responses and the corresponding underlying cortical processes of interest [71, 72]. It is apparent, therefore, that data collected from our sample cannot be directly compared with data from infants or adults since children go through stages of rapid brain growth and development as their neural and cognitive networks reach adulthood [71, 73, 74]. Indeed, resting state simultaneous electroencephalography and fMRI imaging indicates a reduction of recorded signal amplitude between childhood and adulthood [75]. Furthermore, extensive maturation and increased connectivity of sensory neural networks take place during the first few years of life followed by ongoing development and plastic brain changes for a number of years thereafter [76].

In a study conducted by Beauchamp et al. [77], brain-scalp (B-S) distance was investigated in 71 children, from newborn to 12 years, using whole head MRI scans. Differences of up to 50% were found between landmarks, with significantly greater B-S distances observed in frontal and temporal regions, particularly in the RH. B-S distance was also shown to increase with age and in some instances was seen to double from the newborn distance [77]. Since greater B-S distances have been shown to result in more variation in the fNIRS signal [78], it is important to take source and detector distance into consideration to ensure optimal fNIRS recordings [77]. However, the fNIRS system we used only offered a fixed source-detector gap, so, given the expected variation in B-S distance within our sample population, it is possible that even with consistent optode array placement amongst participants the same brain regions were not always being targeted. Furthermore, it is also possible that differences in cerebrospinal fluid volume also influenced the results as increased cerebrospinal fluid results in reduced spatial resolution and a dampened fNIRS signal due to the light scattering characteristics of the fluid [77, 79].

Another important implication from the work carried out by Beauchamp et al. [77] may explain why hemispheric specialisation was not observed in our sample of older children, unlike the left hemispheric dominance for speech described within the infant literature [14, 15, 65, 80]. It has been shown that certain structural asymmetries (e.g., larger temporal gyri and deeper planum temporale) favour the LH from birth [81–86]. Therefore, the reduced B-S distance within the LH in younger children and babies may artificially amplify hemispheric laterality effects for language processing, and the apparent left-lateralisation observed using fNIRS in infants may in fact be artefactual, resulting from variations in B-S distance [77].

However, it is also important to note that the left-lateralisation of speech processing is not always present. For example, Homae et al. [87] found greater activation in right temporoparietal regions in response to normal speech sounds compared to flattened speech sounds in 3 month old infants. Furthermore, in an fNIRS study with adults, Pollonini et al. [54] did not find strong asymmetries between hemispheres in response to various speech stimuli. In fact, they found that their fNIRS measurements were most responsive to activity in the RH which they suggested may be due to responses being elicited from more superficial areas in the RH, making them easier to detect using fNIRS.

When lateralisation effects elicited by normal speech contrasted against TR speech or SCN are present, the presence (or absence) of these hemispheric differences between activation may simply be due to the spectral and temporal variations of the modified signals compared to normal speech. It has been hypothesised that the two cerebral hemispheres preferentially process different aspects of speech due to an underlying acoustic bias (rather than a linguistic bias), such that the left temporal lobe is more specialized for rapid temporal processing, while the right is better at processing spectral information [88–91]. Since TR speech has different temporal characteristics to normal speech [22], it is possible that brain responses and lateralisation effects may be influenced by this. Furthermore, although SCN has a number of features that are acoustically similar to speech and contains the same overall amplitude and spectral profile of the original waveform as well as speech-like rhythmic patterns [16, 24, 92], it still lacks the complexity and richness of speech as all of the spectral detail is replaced with noise [24]. Therefore, again, it is possible that these differences would have impacted responses within each hemisphere differently.

It is interesting to note that under all three auditory stimuli vs silence contrasts, channels showing significant activity were less spread out in the LH than the RH, as shown in the group level cortical activation maps in Fig 2A. This suggests that the speech processing networks in the LH may be more mature and specialised than those within the RH, resulting in a confined language processing centre. This ties in well with the theory that hemispheric dominance arose as a result of interhemispheric conduction delays [93]. This is based on the idea that a faster conduction speed is required when action potentials have to travel greater distances (i.e., in brains which are larger) [94]. Nonetheless, it is important to note that there is evidence to suggest that atypical language lateralisation does not necessarily reflect a disorganised language system or language impairments [95, 96]. In fact, it is worth considering that much of the pre-linguistic and low-level auditory processing is known to engage the auditory cortex bilaterally, with higher level processing then going on to favour the LH [97, 98], which may explain why both TR speech and SCN did not significantly activate the LH as they are both unintelligible speech stimuli.

Although a number of previous studies involving various neuroimaging modalities, including optical imaging techniques, have found significant differences between brain responses elicited by normal vs TR speech contrasts [15, 18, 25], consistent with our findings, there is some strong suggestion in the literature that TR speech is not a good control for normal speech when attempting to identify speech-specific responses [16, 21]. This may be because TR speech is too ‘speech-like’ and is, therefore, processed in a similar way to normal speech [16, 21]. For example, it has been speculated that the left inferior frontal gyrus attempts to process and analyse TR speech as normal speech before it is interpreted as non-linguistic input and the neural response is attenuated in a top-down fashion, resulting in a response pattern that overlaps considerably with that which is produced by normal speech [16]. Similarly, Brown et al. [21] found that TR speech engaged bilateral superior temporal regions more strongly than normal speech, which they claimed was because the temporal reversal of speech does not completely remove intelligibility. Rather, they argued that TR speech results in the perception of ‘confused intelligibility’ rather than ‘removed intelligibility’ [21] with some listeners still able to perceive speech-like features [67].

SCN, on the other hand, is often regarded as a suitable control stimulus when investigating speech processing [16, 20]. Although our findings did not result in such clear conclusions, when investigating activity in posterior superior temporal regions, we did find significantly greater activation in the LH compared to the RH in response to the normal speech vs SCN contrast only. This was due to smaller group averaged ERAs for SCN in the LH than the RH, resulting in a stronger contrast against normal speech. This suggests, at least to some degree, that SCN can offer a stronger contrast to normal speech than TR speech. More interestingly, this indicates that regions closer to Wernicke’s area may be more closely associated with differences in speech intelligibility rather than just low-level auditory processing [99] whereas regions proximal to the primary auditory cortex are more sensitive to the modulation of acoustic stimuli [10]. Since posterior superior temporal regions have also previously been identified as playing a key role in higher level speech processing [67, 99–102] it would prove useful to investigate speech and non-speech responses in this region in more detail. Perhaps the process of isolating speech-specific responses may be clearer within this area of the cortex, especially if the primary AC is more directed towards capturing earlier and general auditory responses [67].

A number of fNIRS studies have successfully identified a relationship between intelligibility and cortical activity, albeit in adult populations. For example, Pollonini et al. [54] found that normal speech elicited the strongest response in the auditory cortex in comparison to the other, less intelligible, speech types used. In a later study, the same group found greater brain activation to normal speech compared to less intelligible speech in NH adults and adult cochlear implant users with good speech perception skills [103]. Defenderfer et al. [104] also found differences in cortical activity in response to easy and more challenging listening conditions. With the possible exception of channel 32, which targeted a more posterior portion of the left auditory cortex, it is possible that the responses measured by the fNIRS system originated from brain regions which respond to any complex, modulated auditory stimulus. Perhaps the fNIRS measurements did not target regions which are specifically sensitive to the intelligibility of the stimulus. For example, a number of studies investigating cortical responses to frequency, amplitude and acoustic modulations, have shown activation in widely distributed sources within primary and secondary auditory cortices [105–111]. As well as this, fMRI data show that a number of different areas within auditory regions in the temporal lobe are active when processing intelligible language [112, 113]. This demonstrates how challenging it can be to draw conclusions about responses elicited by different auditory stimuli when many of the same, overlapping or neighbouring cortical areas are involved.

Given our limited sample size, it is possible that our hypothesised responses were present but that the study was too under-powered to detect them. It is also possible that the responses recorded were influenced by the limitations of the fNIRS system since fNIRS measurements are limited to the outer cortex and parts of the brain that are deeper than approximately 1.5 cm cannot be measured [114]. Therefore, in the present setup, primary auditory cortices would not have been targeted, with measurements likely taken from auditory association regions located in peripheral areas of the temporal lobe. Furthermore, numerous factors, such as the degree of myelination of white matter, optical properties of the scalp, skull, cerebrospinal fluid and hair, as well as source power, for example, can all influence the quality of the fNIRS signal [77, 114]. Additionally, it is essential that a stable optic fibre and scalp contact is maintained throughout the entirety of the imaging duration which can be problematic when testing children [114]. Finally, although the present experimental set-up did not permit this, the use of a multi-distance channel set-up would be beneficial to explore in future work.

Nonetheless, it is surprising not to observe any difference between speech and non-speech stimulation in our participant cohort. If fNIRS is to be considered as a technique for use in clinical settings to identify successful speech signal recognition and speech-specific processing, we would require at least some effects to be present in 23 NH children if the metric is sensitive and specific enough to be used at an individual level in clinical populations.

## Conclusion

Neither TR speech nor SCN appear to be suitable baselines for functionally isolating speech-specific processing in an experimental set up involving fNIRS with 6–12 year old NH children. We did not observe differences in cortical activation patterns between the two brain hemispheres elicited by the different stimuli contrasts even at a group level. Our participant sample consisted of an age group known to be undergoing extensive brain development and who exhibit a high degree of individual variability, which may help to explain why no effects were found. It is also possible that the limited spatial resolution and low cortical depth penetration of fNIRS may have contributed towards the substantial overlap between responses to normal speech, TR speech and SCN. It is important to continue investigations in this area to develop effective procedures for high quality non-invasive imaging of auditory language function. The appropriateness of other auditory baselines for isolating speech-specific activity should be considered in future work.

## Supporting information

S1 File Study data. The beta values underlying the findings described in the manuscript.
(XLSX)
